# P-764. Epidemiology of Nontuberculosis *Mycobacteria* (NTM) in Monroe County, NY: 2021-2023

**DOI:** 10.1093/ofid/ofae631.959

**Published:** 2025-01-29

**Authors:** christopher J Myers, Ghinwa Dumyati

**Affiliations:** University of Rochester, Rochester, New York; New York Emerging Infections Program and University of Rochester Medical Center, Rochester, New York

## Abstract

**Background:**

Nontuberculosis mycobacteria (NTM) are opportunistic pathogens causing complex infections requiring prolonged multi-drug therapy and/or surgical interventions. The incidence rates of NTM are increasing nationally, however NTM is currently not a reportable in many states, and likely underreported. To provide a better understanding the burden of NTM, we describe the epidemiology over a 3 year surveillance period.

**Figure:**
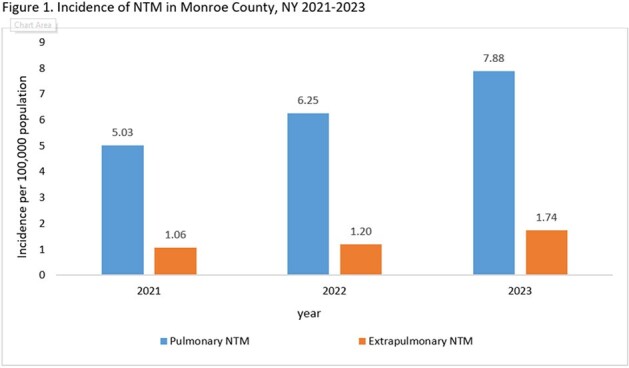

**Methods:**

Active laboratory and population-based surveillance was conducted in Monroe County, NY as part of the CDC Emerging Infections Program. Demographic, clinical, and laboratory data were collected via medical record review. Cases were categorized as pulmonary (PNTM) or extrapulmonary (ENTM) based on source of isolation, with PNTM further categorized into possible or confirmed. A case was defined as incident if NTM was not isolated in the 12 months prior to the positive culture, or prevalent if NTM was present in the prior 12 months. Incidence rates were calculated for 2021-2023 using U.S. Census population estimates, and descriptive analyses were performed to describe demographic and clinical characteristics of PNTM vs ENTM cases.

**Figure:**
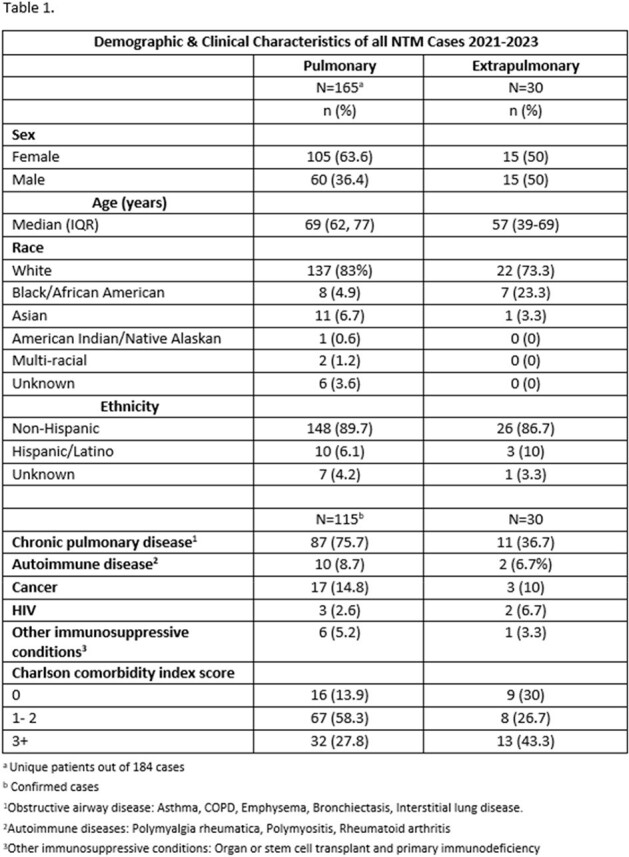

**Results:**

Incidence rates of PNTM and ENTM increased from 5.3 and 1.06 to 7.88 and 1.74 per 100,000 population between 2021-2023, respectively (Figure 1). PNTM and ENTM cases were both predominantly White (83% vs 73%), Non-Hispanic (90% vs 87%). PNTM cases were older (Median 69 yrs; IQR: 62, 77) than ENTM cases (Median 57yrs, IQR: 39, 69) and more likely to have underlying obstructive airway disease (Table 1). *M. avium* complex was most common in PNTM cases and rapid growing NTM species in ENTM cases (Table 2). Notably 50% of PNTM cases and 70% of ENTM cases had a clinical diagnosis of NTM disease. ENTM cases had trauma (13%), surgery (17%), medical devices (27%) or injections (13%) at the site of infection.

**Figure:**
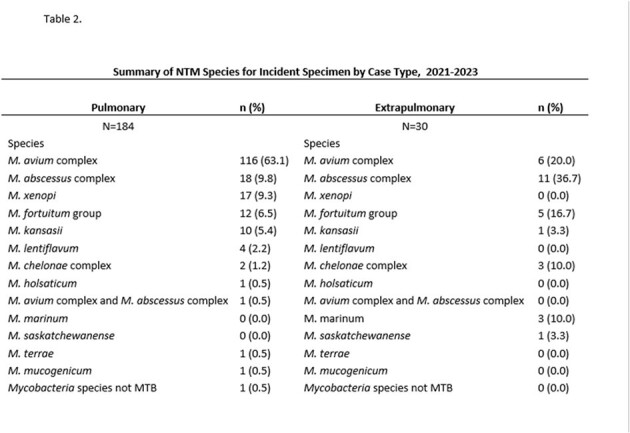

**Conclusion:**

This study reveals a rising incidence of both PNTM and ENTM infections with variations in species distribution and associated risk factors for ENTM such as surgical procedures and medical devices. These results underscore the need for ongoing monitoring and targeted interventions to address the growing public health burden of NTM infections.

**Disclosures:**

**All Authors**: No reported disclosures

